# Circ_0001947 promotes cell proliferation, invasion, migration and inflammation and inhibits apoptosis in human rheumatoid arthritis fibroblast-like synoviocytes through miR-671-5p/STAT3 axis

**DOI:** 10.1186/s13018-022-02939-3

**Published:** 2022-01-29

**Authors:** Yang Yang, Shudian Lin, Zhou Yang, Yanyan Huang, Feng Zhan

**Affiliations:** Department of Rheumatology & Immunology, Hainan General Hospital/Hainan Affiliated Hospital of Hainan Medical University, No. 19 Xiuhua Road, Xiuying District, Haikou City, 570311 Hainan Province China

**Keywords:** RA-FLSs, circ_0001947, miR-671-5p, STAT3

## Abstract

**Background:**

Circular RNAs (circRNAs) have emerged as vital regulators in the development of rheumatoid arthritis (RA). In this study, we aimed to explore the functions and mechanisms of circ_0001947 in RA.

**Methods:**

The expression of circ_0001947, microRNA-671-5p (miR-671-5p) and signal transducer and activator of transcription 3 (STAT3) was determined by quantitative real-time polymerase chain reaction (qRT-PCR) or western blot. Cell Counting Kit-8 (CCK-8) assay, 5′-ethynyl-2′-deoxyuridine (EdU) assay, flow cytometry analysis, transwell assay and wound-healing assay were performed to assess cell proliferation, apoptosis, invasion and migration. The concentrations of inflammatory factors were examined with enzyme-linked immunosorbent assay (ELISA) kits. Dual-luciferase reporter assay was used to analyze the relationships of circ_0001947, miR-671-5p and STAT3.

**Results:**

Circ_0001947 was upregulated in RA patients and RA-FLSs. Knockdown of circ_0001947 repressed cell proliferation, invasion, migration and inflammatory response and facilitated apoptosis in RA-FLSs. Circ_0001947 served as the sponge for miR-671-5p and the inhibitory effect of circ_0001947 in RA-FLS progression was reversed by miR-671-5p inhibition. STAT3 was the target gene of miR-671-5p. MiR-671-5p overexpression restrained RA-FLS growth, invasion, migration and inflammation and promoted apoptosis, but STAT3 upregulation reversed the impacts.

**Conclusion:**

Circ_0001947 contributed to the progression of RA-FLSs by elevating STAT3 through adsorbing miR-671-5p.

## Highlights

1. Circ_0001947 is abnormally upregulated in RA synovial tissues and RA-FLSs.
2. Circ_0001947 silencing inhibits cell proliferation, invasion, migration and inflammatory response and induces apoptosis in RA-FLSs.
3. Circ_0001947 sponges miR-671-5p, which directly interacts with STAT3.
4. Circ_0001947 regulates RA-FLS progression via miR-671-5p/STAT3 axis.

## Introduction

Rheumatoid arthritis (RA) is a chronic autoimmune disease and featured by the progressive damage to cartilage and bone, imposing a huge burden on patients and society [1, 9, 27]. Synovial tissues are the membranous organ within the articular lumen and play a role in joint destruction in RA [16]. As the key cells related to synovial tissue formation, fibroblast-like synovial cells (FLSs) are involved in the inflammatory response of synovial joints, leading to cartilage destruction and RA [14]. In addition, RA-FLSs have been reported to have the similar properties to tumor cells, such as proliferation, invasion, migration, and anti-apoptosis [3, 25]. Therefore, exploring the pathological mechanism of FLSs in RA may be helpful to find novel therapeutic methods for RA.

Circular RNAs (circRNAs) are covalently closed non-coding RNAs (ncRNAs) and play vital roles in the biological processes in human diseases [15, 21]. Currently, the involvement of circRNAs in RA has been reported [28]. It has been widely documented that circRNAs can sponge microRNAs (miRNAs) and then elevate the stability of mRNAs [19, 35]. In RA, circ_0000396 curbed RA-FLS growth and inflammation and aggravated apoptosis via decoying miR-203 and upregulating HBP1 [30]. Circ_0088036 contributed to RA-FLS growth and migration by miR-140-3p/SIRT1 axis [34]. Circ_0001947 (also termed as circAFF2) was derived from AFF2 and has been demonstrated to regulate the inflammatory response of RA-FLSs through miR-650/CNP pathway [23]. Even so, circ_0001947-medaited mechanisms in RA progression are little known.

MiRNAs are short ncRNAs that can hinder gene expression by interacting with the 3′UTR of target mRNAs [24]. MiRNAs have been reported to act as vital regulators in human diseases, such as tendon injuries [8, 10] and osteoarthritis [22]. Moreover, the relation between miRNAs and RA is widely identified [20]. For instance, miR-27a-3p [6], miR-499 [11] and miR-140-3p [38] could regulate FLS growth, invasion, apoptosis and inflammation by targeting TLR5, HDAC1 or SIRT3. MiR-671-5p was linked to the progression of diverse human diseases, such as ischemic stroke [7], gastric cancer [26] and osteosarcoma [17]. More importantly, miR-671-5p participated in the modulation of FLS malignant changes in RA [5].

Signal transducer and activator of transcription 3 (STAT3) is a member of STAT family and the activation of STAT3 pathway is related to RA-FLS progression [32]. Zhu et al. declared that miR-140-5p restrained the growth and inflammation and enhanced the apoptosis of RA-FLSs by targeting STAT3 [37]. Even so, the relation between miR-671-5p and STAT3 is unclear.

In the present research, the functions and mechanisms of circ_0001947, miR-671-5p and STAT3 in the regulation of RA-FLS progression were investigated.

## Materials and methods

### Clinical samples

The RA synovial tissues were acquired from RA patients (17 males and 12 females; aged 38–62) who suffered from knee joint replacement surgery at Hainan General Hospital and the normal control synovial tissues were collected from the patients (15 males and 14 females, aged 36–59) who underwent emergency trauma amputation. All samples were recruited during May 2016 and March 2021. Patients who had a history of joint abnormalities, systemic diseases, autoimmune or infectious diseases were excluded from the normal group. The approval was obtained from the Ethics Committee of Hainan General Hospital. Written informed consents were signed by the participants.

### RA-FLS culture

The RA-FLSs and normal FLSs were isolated from the synovial tissues of RA patients and normal controls. In brief, the samples were cut into small pieces and then digested with 0.1% collagenase type I (Invitrogen, Carlsbad, CA, USA) for 4 h. After 5 min of centrifugation at 1000×, the cells were obtained and cultured in DMEM (Invitrogen) containing 10% FBS (Invitrogen) and 1% penicillin–streptomycin (Invitrogen) under the conditions of 37 °C and 5% CO_2_. The cells at passages 3–5 were used for further experiments.

### Quantitative real-time polymerase chain reaction (qRT-PCR)

Total RNA in tissues and FLSs was isolated using TRIzol reagent (Invitrogen) and cDNA synthesis was conducted on the RNA using M-MLV (Promega, Madison, WI, USA) or TaqMan MicroRNA Reverse Transcription reagent (Applied Biosystems, Foster City, CA, USA). The amplification of cDNAs was conducted using SYBR Premix DimerEraser (Takara, Dalian, China). The primers were exhibited in Table 1. The expression was computed through the 2^−ΔΔCt^ strategy with normalization to GAPDH or U6.

**Table 1:** Primers sequences used for qRT-PCR

Name		Primers for PCR (5′–3′)
circ_0001947	Forward	ACACTCTTGGATGGAAAACCCA
	Reverse	CGTGTTCTGGACTCGGTTGG
AFF2	Forward	CTGACAGCGAATCTAATGAGGC
	Reverse	CATTGGTTGGATGATTGGAGGA
miR-671-5p	Forward	AGGAAGCCCTGGAGGG
	Reverse	GAACATGTCTGCGTATCTC
STAT3	Forward	CAGCAGCTTGACACACGGTA
	Reverse	AAACACCAAAGTGGCATGTGA
GAPDH	Forward	AGCTCACTGGCATGGCCTTC
	Reverse	CGCCTGCTTCACCACCTTCT
U6	Forward	GCTTCGGCAGCACATATACTAA
	Reverse	AACGCTTCACGAATTTGCGT

### RNase R treatment

To analyze the stability of circ_0001947, the RNA in RA-FLSs was treated with or without RNase R (Epicenter, Madison, WI, USA). Then the enrichment of circ_0001947 and AFF2 was quantified.

### Subcellular fraction analysis

The PARIS reagent (Ambion, Austin, TX, USA) was utilized to separate the nuclear and cytosolic fractions in RA-FLSs referring to the manufacturers’ instructions. Then the RNAs isolated from the fractions were subjected to qRT-PCR to examine the levels of circ_0001946, GAPDH (cytoplasmic control transcript) and U6 (nuclear control transcript).

### Cell transfection

Small interfering RNA against circ_0001947 (si-circ_0001947) and scramble control si-NC, circ_0001947 overexpression vector (circ_0001947) and its control (pCD5-ciR), miR-671-5p mimics and inhibitors (miR-671-5p and anti-miR-671-5p) and related controls miR-NC and anti-miR-NC, STAT3 overexpression vector (STAT3) and empty control (pcDNA) were synthesized by GenePharma (Shanghai, China). RA-FLSs were plated into 6-well plates and then transfected with the compositions using Lipofectamine 2000 (Invitrogen).

### Cell Counting Kit-8 (CCK-8) assay

To examine RA-FLS viability, RA-FLSs were cultured overnight in 96-well plates and then added 10 μL CCK-8 (Beyotime, Shanghai, China) for further incubation for 4 h. The absorption at 450 nm was examined by a microplate reader (Bio-Rad, Hercules, CA, USA).

### 5′-ethynyl-2′-deoxyuridine (EdU) analysis

By using EdU assay reagent (RIBOBIO, Guangzhou, China), RA-FLS proliferation was analyzed. Shortly, RA-FLSs were seeded into 12-well plates and then kept for 2 h with EdU. After that, the cells were fixed in paraformaldehyde (Sigma-Aldrich, St. Louis, MO, USA), kept with 0.5% Triton-X-100 (Sigma-Aldrich) and then stained with Apollo and DAPI. The images were acquired using a fluorescence microscope (Olympus, Tokyo, Japan).

### Flow cytometry analysis

RA-FLS apoptosis was examined with Annexin V-fluorescein isothiocyanate (FITC)/propidium iodide (PI) reagent (Beyotime). In short, RA-FLSs were resuspended in binding buffer and dual-labeled with Annexin V-FITC and PI for 20 min without light. The flow cytometer (Beckman Coulter, Brea, CA, USA) was used to estimate the apoptotic rate.

### Transwell assay

To estimate RA-FLS invasion, the Matrigel (Corning, Corning, NY, USA)-coated transwell chambers (Corning) were adopted. RA-FLSs in serum-free DMEM were added into the upper chambers and DMEM containing 10% FBS was added into the lower chambers. After 24 h, RA-FLSs passed through the membranes were stained with crystal violet (Sigma-Aldrich) and imaged under a microscope (100×; Olympus).

### Wound-healing assay

For the evaluation of RA-FLS migration, RA-FLSs were grown in 12-well plates until 90% confluence and then a new pipette tip was used to make the scratch on the plates. The scratch width was recorded at 0 h and 24 h.

### Western blot assay

The proteins were extracted using RIPA buffer (Beyotime) and then separated via 10% SDS-PAGE electrophoresis. Next, the samples were electroblotted onto PVDF membranes and blocked for 2 h in 5% slim milk. Thereafter, the membranes were probed with primary antibodies against Cyclin D1 (ab226977, Abcam, Cambridge, MA, USA), MMP9 (ab228402, Abcam), STAT3 (ab68153, Abcam) and GAPDH (ab9485, Abcam) followed by incubation with secondary antibody (ab6789, Abcam). The protein bands were visualized using the ECL kit (Beyotime).

### Enzyme-linked immunosorbent assay (ELISA)

The concentrations of TNF-α and IL-1β in the supernatants of RA-FLSs were measured using TNF-α and IL-1β ELISA kits (ab181421, ab2140255, Abcam) in line with the protocols.

### Dual-luciferase reporter assay

The sequences of wild-type (WT) circ_0001947 or STAT3 3′UTR containing miR-671-5p binding sites and related mutant (MUT) lacking miR-671-5p binding sites were cloned into pmirGLO vector (Promega, Fitchburg, WI, USA) to generate dual-luciferase reporter vectors WT-circ_0001947, WT-STAT3 3**′**UTR, MUT-circ_0001947 and MUT-STAT3 3**′**UTR, respectively. The vectors and miR-NC/miR-671-5p were then co-transfected into RA-FLSs followed by measurement of the luciferase intensity with dual-Luciferase Reporter Assay Reagent (Promega).

### Statistical analysis

GraphPad Prism 7 was used to analyze the data. The results were exhibited as mean ± SD. The experiments were conducted triple times. The differences in different groups were estimated via Student’s *t*-test or one-way ANOVA. Spearman’s correlation coefficient was utilized for linear correlation analysis. *P* < 0.05 was considered as significant.

## Results

### Circ_0001947 was overexpressed in RA synovial tissues and RA-FLSs

**Fig. 1:**
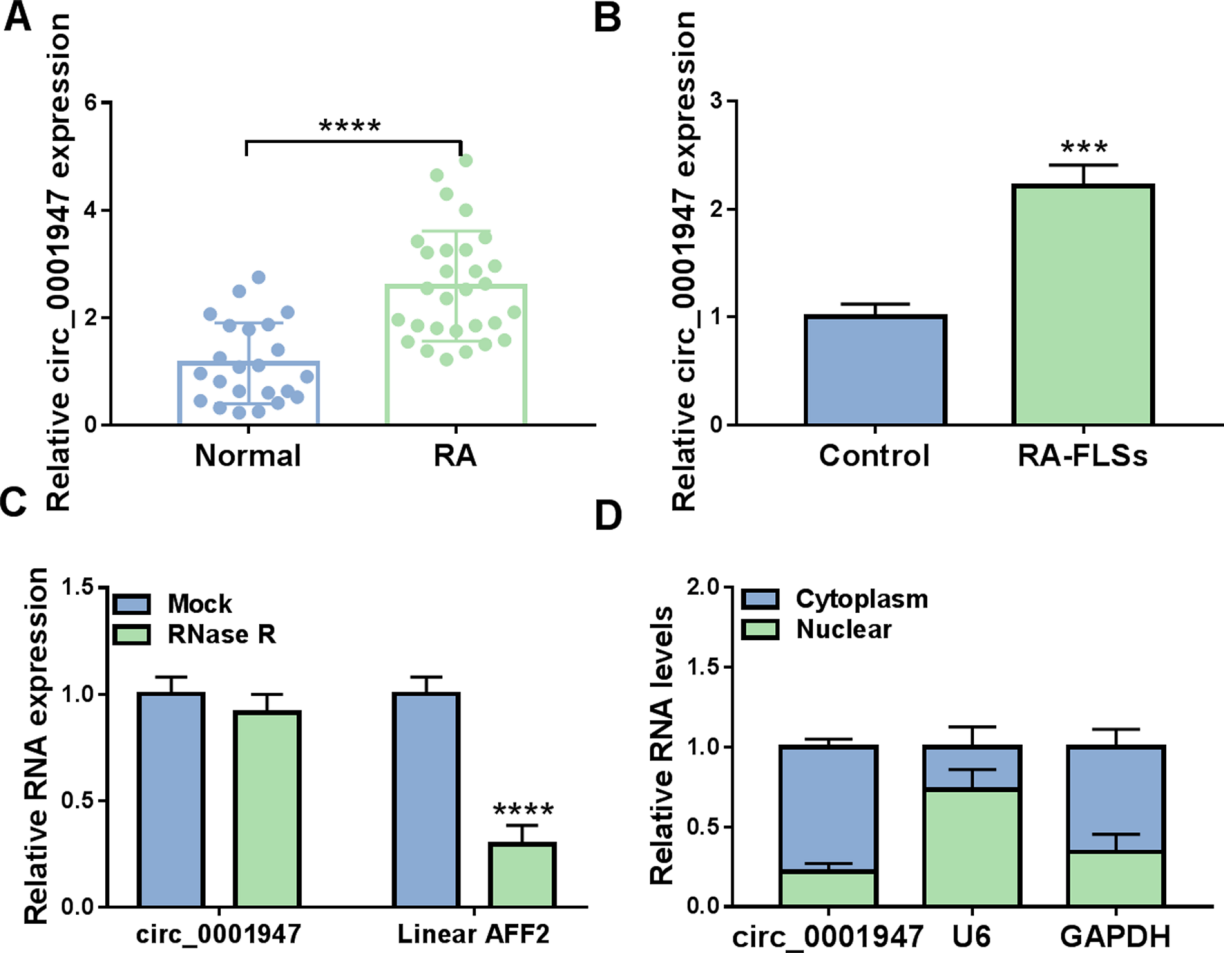
Circ_0001947 was upregulated in RA synovial tissues and RA-FLSs. **A** The expression of circ_0001947 in the synovial tissues of RA patients and normal controls was detected by qRT-PCR. **B** The expression of circ_0001947 in RA-FLSs and normal FLSs was detected by qRT-PCR. **C** After treatment with RNase R, the expression levels of circ_0001947 and AFF2 in RA-FLSs were determined by qRT-PCR. **D** The expression of circ_0001947 in the cytoplasm and nuclear of RA-FLSs was detected by qRT-PCR. ***P < 0.001, ****P < 0.0001

As shown in Fig. 1A, circ_0001947 was highly expressed in the synovial tissues of RA patients compared to normal synovial tissues. Compared to normal FLSs, circ_0001947 level was increased in RA-FLSs (Fig. 1B). RNase R assay indicated that circ_0001947 could not be digested by RNase R, while linear AFF2 was markedly digested by RNase R (Fig. 1C). The results indicated that circ_0001947 was more stable than linear AFF2. Then we found that circ_0001947 was mainly enriched in the cytoplasm (Fig. 1D).

### Circ_0001947 knockdown suppressed cell proliferation, invasion, migration and inflammation and promoted apoptosis in RA-FLSs

**Fig. 2:**
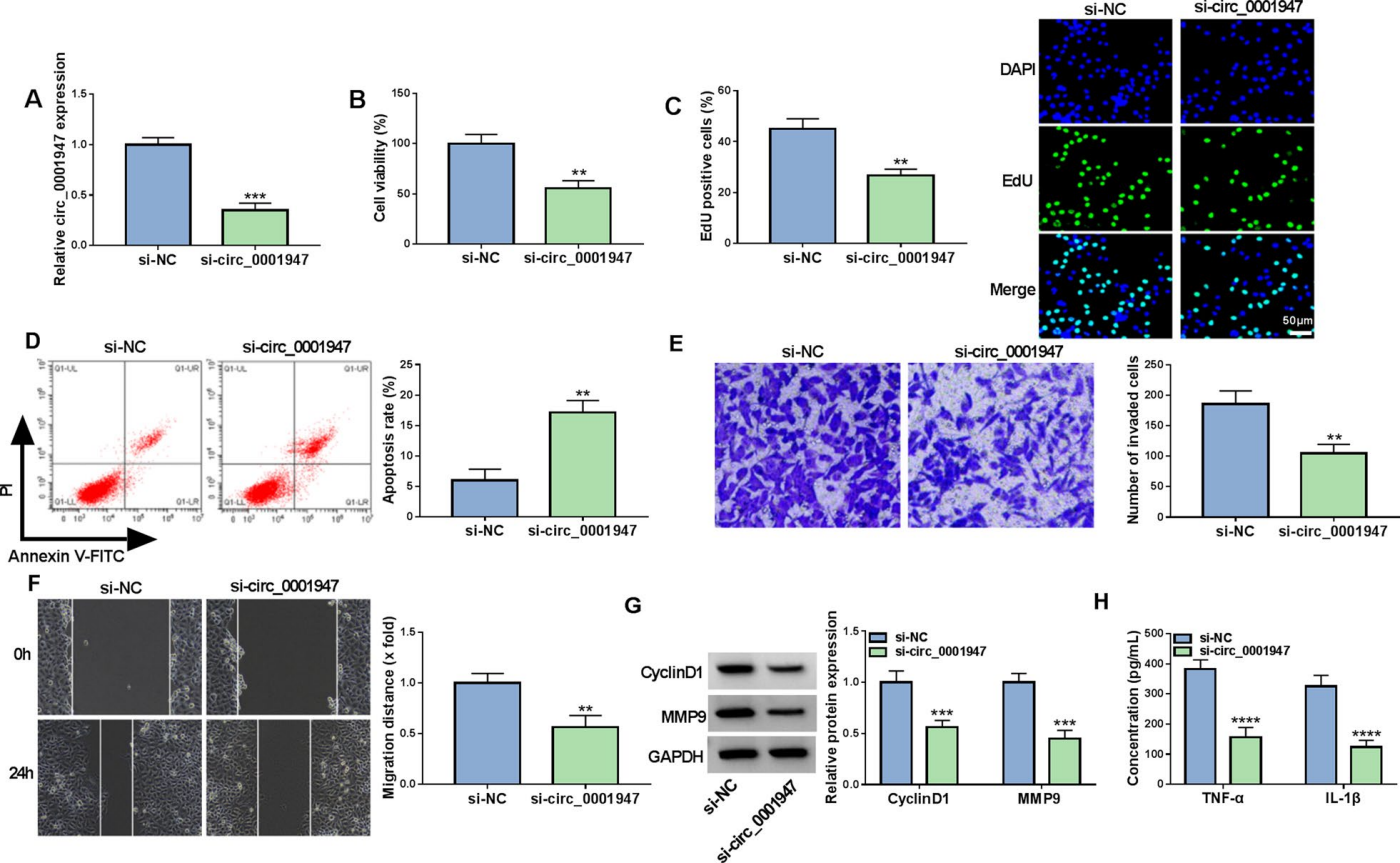
Effects of circ_0001947 on RA-FLS cell growth, apoptosis, invasion, migration and inflammation. RA-FLSs were introduced with si-NC or si-circ_0001947. **A** The expression of circ_0001947 in RA-FLSs was examined by qRT-PCR. **B**, **C** The proliferation of RA-FLSs was estimated by CCK-8 assay and EdU assay. **D** The apoptosis of RA-FLSs was analyzed by flow cytometry analysis. **E**, **F** The invasion and migration of RA-FLSs were assessed by transwell assay and wound-healing assay. **G** The protein levels of Cyclin D1 and MMP9 in RA-FLSs were measured via western blot assay. **H** The concentrations of TNF-α and IL-1β in RA-FLSs were examined by ELISA. **P < 0.01, ***P < 0.001, ****P < 0.0001

To explore the function of circ_0001947 in RA development, RA-FLSs were transfected with si-circ_0001947 to knock down circ_0001947 expression. The results were identified by qRT-PCR, which showed that si-circ_0001947 transfection evidently decreased circ_0001947 expression in RA-FLSs compared to si-NC control group (Fig. 2A). As illustrated by CCK-8 assay and EdU assay, the proliferation of RA-FLSs was repressed by silencing circ_0001947 (Fig. 2B, C). Flow cytometry analysis indicated that circ_0001947 interference facilitated the apoptosis of RA-FLSs in comparison with si-NC control group (Fig. 2D). Transwell assay showed that circ_0001947 deficiency inhibited the RA-FLSs to invade compared to si-NC control group (Fig. 2E). Wound-healing assay exhibited that circ_0001947 knockdown repressed RA-FLS migration (Fig. 2F). Moreover, circ_0001947 knockdown reduced the protein levels of Cyclin D1 and MMP9 in RA-FLSs in comparison with si-NC control group (Fig. 2G). In addition, circ_0001947 knockdown decreased the concentrations of TNF-α and IL-1β in RA-FLSs (Fig. 2H). These findings suggested that circ_0001947 knockdown inhibited the progression of RA-FLSs.

### Circ_0001947 sponged miR-671-5p

**Fig. 3:**
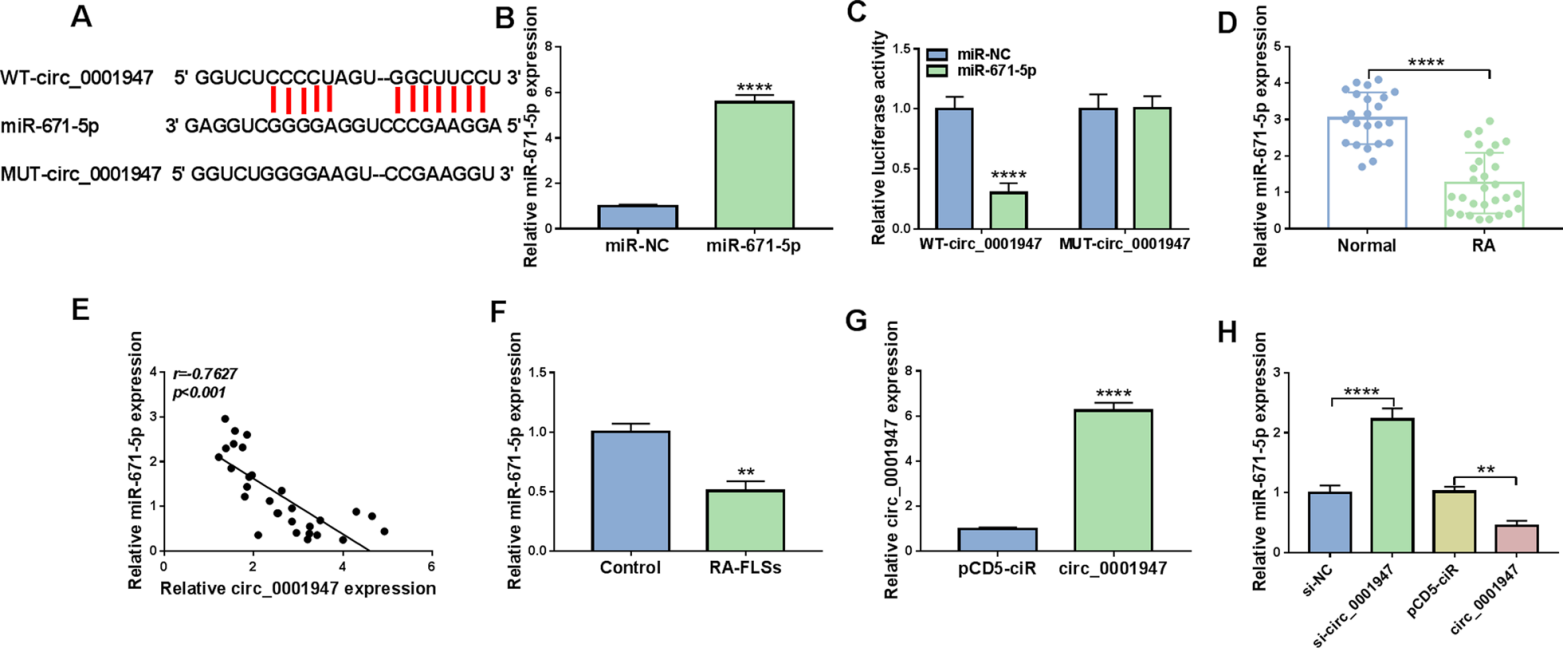
MiR-671-5p was the target of circ_0001947. **A** The binding sites between circ_0001947 and miR-671-5p. **B** The expression of miR-671-5p in RA-FLSs transfected with miR-NC or miR-671-5p was detected by qRT-PCR. **C** The relationship between circ_0001947 and miR-671-5p was demonstrated by dual-luciferase reporter assay. **D** The expression of miR-671-5p in RA patients and normal controls was determined by qRT-PCR. **E** The correlation between the levels of circ_0001947 and miR-671-5p in RA patients was analyzed. **F** The expression of miR-671-5p in RA-FLSs and normal FLSs was detected by qRT-PCR. **G** The expression of circ_0001947 in RA-FLSs transfected with pCD5-ciR or circ_0001947 was examined via qRT-PCR. **H** The expression of miR-671-5p in RA-FLSs transfected with si-NC, si-circ_0001947, pCD5-ciR or circ_0001947 was detected by qRT-PCR. **P < 0.01, ****P < 0.0001

Through analyzing the bioinformatics prediction website circinteractome (https://circinteractome.irp.nia.nih.gov/), we found that miR-671-5p contained the binding sites of circ_0001947 (Fig. 3A). The mimics of miR-671-5p transfection drastically increased miR-671-5p expression in RA-FLSs (Fig. 3B). Then dual-luciferase reporter assay showed that miR-671-5p overexpression repressed the luciferase activity of WT-circ_0001947 in RA-FLSs but not MUT-circ_0001947, suggesting the combination between circ_0001947 and miR-671-5p (Fig. 3C). Indeed, miR-671-5p level was decreased in RA synovial tissues and negatively correlated with circ_0001947 level (Fig. 3D, E). Compared to normal FLSs, miR-671-5p was lowly expressed in RA-FLSs (Fig. 3F). The transfection of circ_0001947 overexpression vector led to an elevation in circ_0001947 expression in RA-FLSs compared to pCD5-ciR vector (Fig. 3G). Of note, circ_0001947 knockdown increased miR-671-5p expression in RA-FLSs, while circ_0001947 overexpression decreased miR-671-5p (Fig. 3H). Taken together, circ_0001947 sponged miR-671-5p to negatively regulate miR-671-5p expression.

### Circ_0001947 knockdown regulated cell proliferation, apoptosis, invasion, migration and inflammation in RA-FLSs by targeting miR-671-5p

**Fig. 4:**
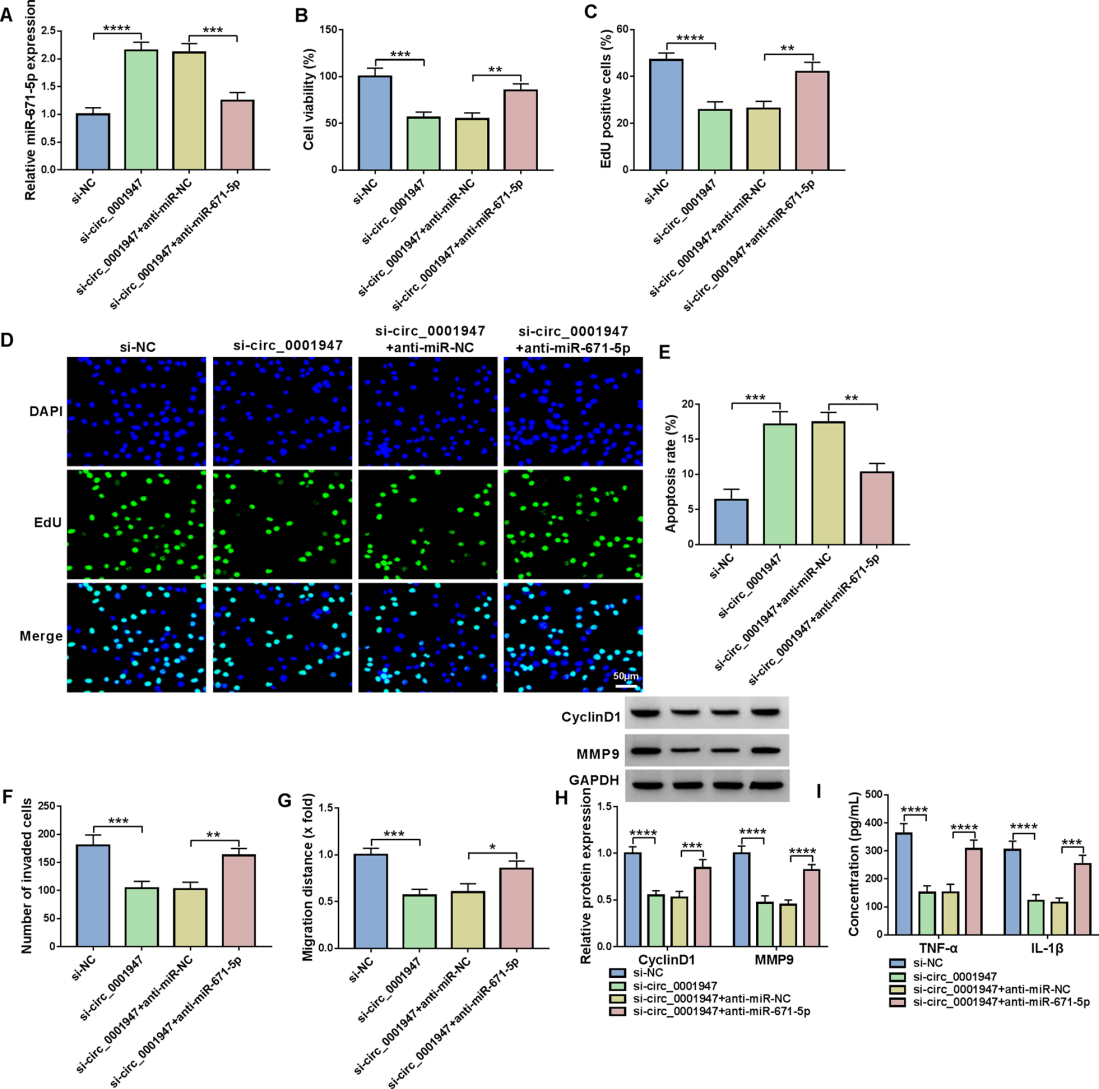
Circ_0001947 regulated RA-FLS progression by sponging miR-671-5p. RA-FLSs were transfected with si-NC, si-circ_0001947, si-circ_0001947 + anti-miR-NC or si-circ_0001947 + anti-miR-671-5p. **A** The expression of miR-671-5p in RA-FLSs was detected by qRT-PCR. **B**–**D** The proliferation of RA-FLSs was examined via CCK-8 assay and EdU assay. **E**–**G** The apoptosis, invasion and migration of RA-FLSs were estimated by flow cytometry analysis, transwell assay and wound-healing assay, respectively. **H** The protein levels of CyclinD1 and MMP9 in RA-FLSs were measured by western blot assay. **I** The concentrations of TNF-α and IL-1β in RA-FLSs were examined by ELISA kits. *P < 0.05, **P < 0.01, ***P < 0.001, ****P < 0.0001

To explore the relationship between circ_0001947 and miR-671-5p in regulating RA-FLS progression, rescue experiments were performed. As shown in Fig. 4A, circ_0001947 silencing-caused upregulation of miR-671-5p expression in RA-FLSs was reversed by anti-miR-671-5p transfection. The results of CCK-8 and EdU assay showed that the inhibitory effect of circ_0001947 knockdown on RA-FLS proliferation was abated by decreasing miR-671-5p (Fig. 4B–D). Flow cytometry analysis showed that circ_0001947 knockdown facilitated RA-FLS apoptosis, while miR-671-5p inhibition reversed the facilitation (Fig. 4E). As demonstrated by transwell assay and wound-healing assay, circ_0001947 knockdown restrained the invasion and migration of RA-FLSs, with miR-671-5p inhibition ameliorated the impacts (Fig. 4F, G). The reduction of CyclinD1 and MMP9 levels in RA-FLSs transfected with si-circ_0001947 was also rescued by decreasing miR-671-5p (Fig. 4H). In addition, circ_0001947 knockdown decreased the concentrations of TNF-α and IL-1β in RA-FLSs, whereas miR-671-5p inhibition rescued the effects (Fig. 4I). Collectively, circ_0001947 knockdown inhibited RA-FLS progression by targeting miR-671-5p.

### MiR-671-5p interacted with STAT3

**Fig. 5:**
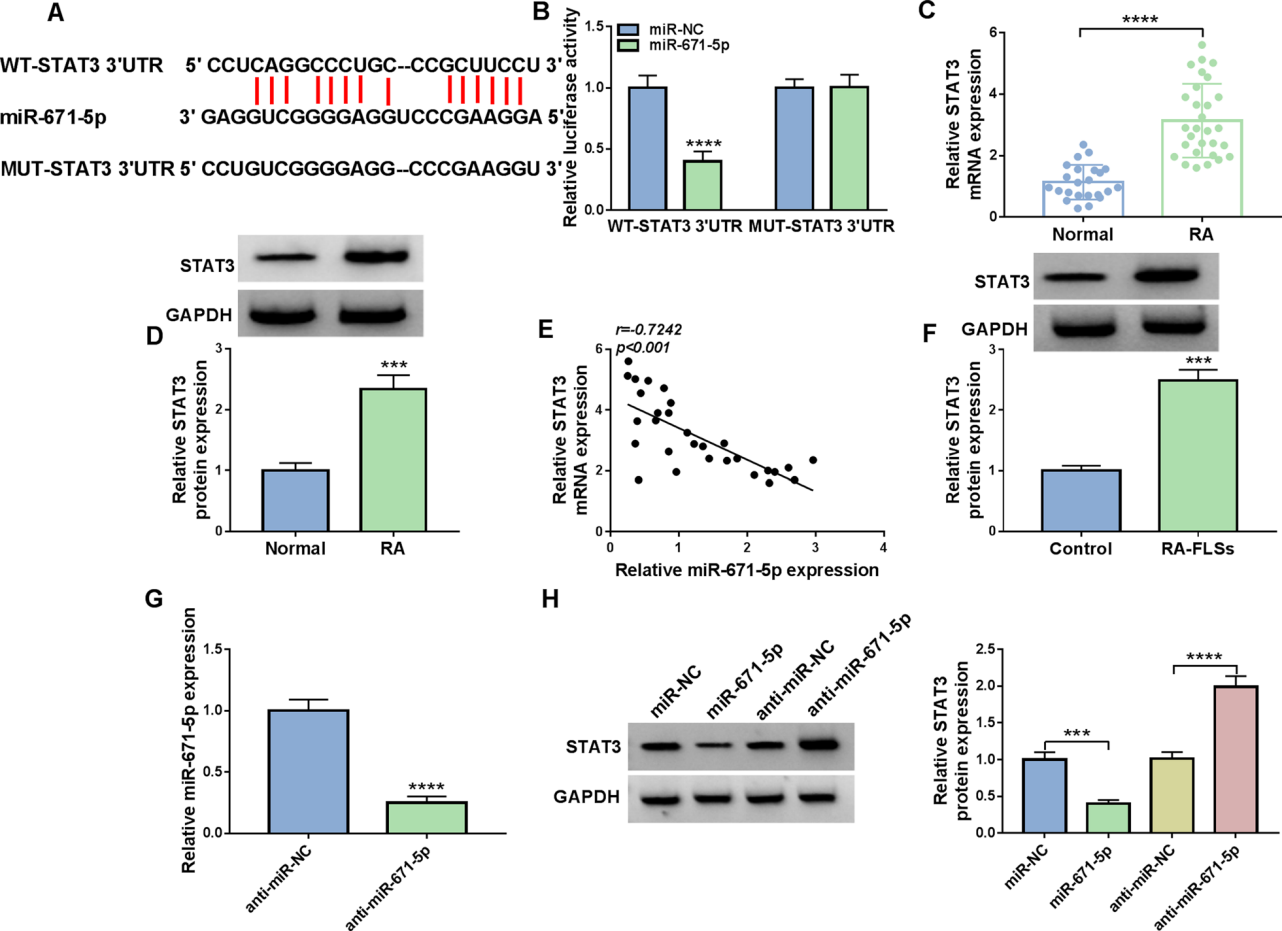
MiR-671-5p directly targeted STAT3. **A** The binding sites between STAT3 and miR-671-5p. **B** The association between miR-671-5p and STAT3 was verified by dual-luciferase reporter assay. **C**, **D** The mRNA and protein levels of STAT3 in RA and normal synovial tissues were measured via qRT-PCR and western blot, respectively. **E** The linear correlation between STAT3 and miR-671-5p in RA synovial tissues was analyzed. **F** The protein level of STAT3 in RA-FLSs and normal FLSs was measured by western blot. **G** The expression of miR-671-5p in RA-FLSs transfected with anti-miR-NC or anti-miR-671-5p was detected by qRT-PCR. **H** The protein level of STAT3 in RA-FLSs transfected with miR-NC, miR-671-5p, anti-miR-NC or anti-miR-671-5p was measured by western blot. ***P < 0.001, ****P < 0.0001

Through analyzing starBase (http://starbase.sysu.edu.cn/), STAT3 was found to be the target gene of miR-671-5p (Fig. 5A). Dual-luciferase reporter assay further demonstrated the interaction between STAT3 and miR-671-5p for the luciferase activity of WT-STAT3 3′UTR was reduced after miR-671-5p overexpression, but the luciferase activity of MUT-STAT3 3′UTR was not changed (Fig. 5B). Compared to normal synovial tissues, STAT3 mRNA and protein levels were upregulated in RA synovial tissues (Fig. 5C, D). Moreover, there was an inverse correlation between the levels of STAT3 and miR-671-5p in RA synovial tissues (Fig. 5E). STAT3 protein level was increased in RA-FLSs compared to normal controls (Fig. 5F). The transfection of anti-miR-671-5p markedly reduced miR-671-5p expression in RA-FLSs compared to anti-miR-NC control groups (Fig. 5G). Besides, miR-671-5p overexpression reduced STAT3 protein level in RA-FLSs, but miR-671-5p inhibition elevated STAT3 protein level (Fig. 5H). To summarize, miR-671-5p inhibited STAT3 expression by targeting STAT3.

### Overexpression of miR-671-5p suppressed cell proliferation, invasion, migration and inflammation and induced apoptosis in RA-FLSs by targeting STAT3

**Fig. 6:**
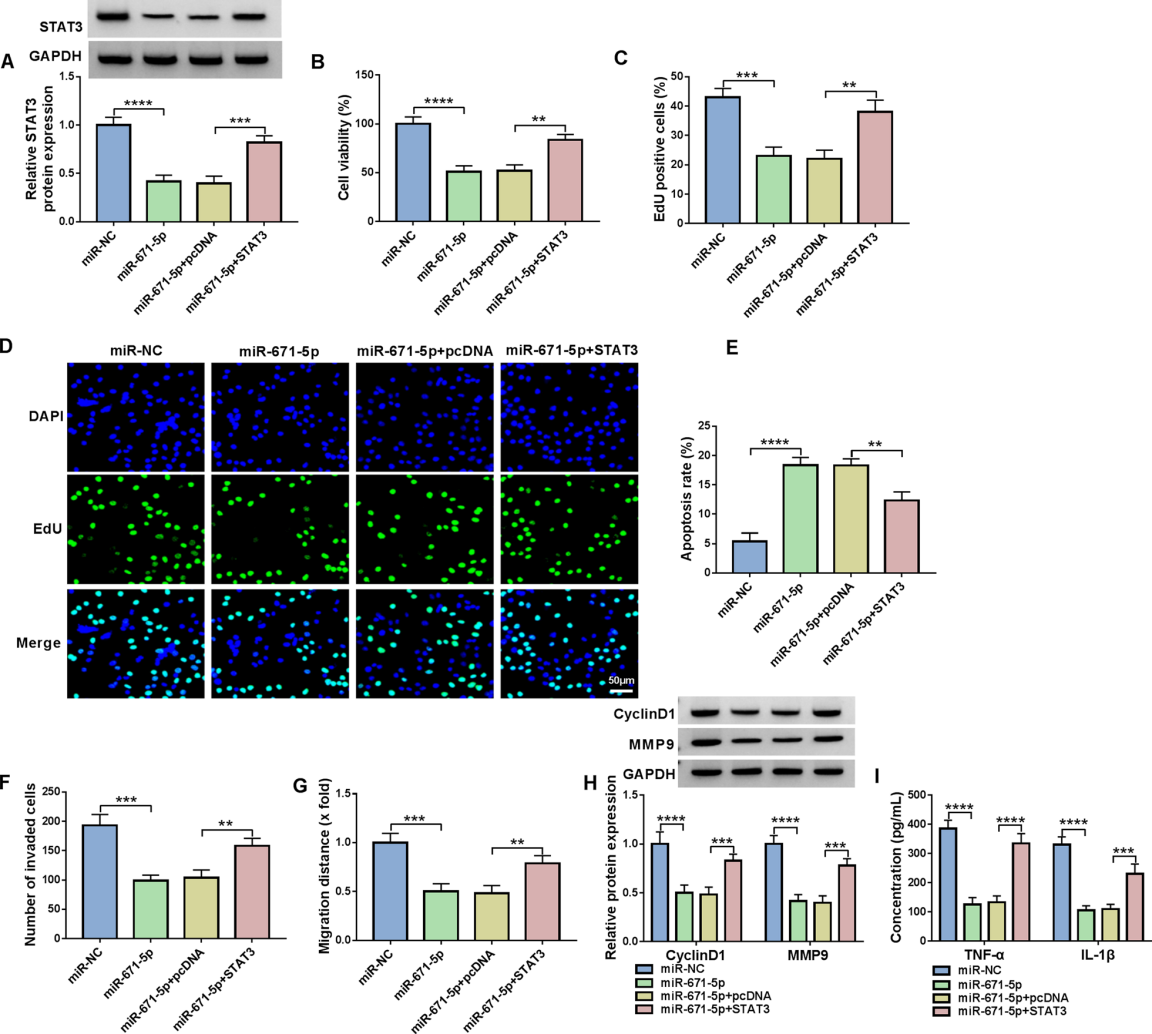
MiR-671-5p inhibited the progression of RA-FLSs by interacting with STAT3. RA-FLSs were transfected with miR-NC, miR-671-5p, miR-671-5p + pcDNA or miR-671-5p + STAT3. **A** The protein level of STAT3 in RA-FLSs was measured by western blot assay. **B**–**D** The proliferation of RA-FLSs was evaluated by CCK-8 assay and EdU assay. **E** The apoptosis of RA-FLSs was assessed by flow cytometry analysis. **F**, **G** The invasion and migration of RA-FLSs were examined with transwell assay and wound-healing assay, respectively. **H** The protein levels of CyclinD1 and MMP9 in RA-FLSs were measured by western blot assay. **I** The concentrations of TNF-α and IL-1β in RA-FLSs were examined with ELISA kits. **P < 0.01, ***P < 0.001, ****P < 0.0001

Subsequently, the relationship between miR-671-5p and STAT3 in RA-FLSs progression was investigated. As presented in Fig. 6A, miR-671-5p mimic transfection reduced STAT3 protein level in RA-FLSs, while the reduction was relieved by STAT3 overexpression vector transfection. CCK-8 assay and EdU assay exhibited that miR-671-5p restrained the proliferation of RA-FLSs, while the effect was weakened by upregulating STAT3 (Fig. 6B–D). Flow cytometry analysis indicated that the apoptosis of RA-FLSs was facilitated by miR-671-5p elevation but further reversed by enhancing STAT3 (Fig. 6E). Transwell assay and wound-healing assay indicated that miR-671-5p overexpression suppressed the invasion and migration of RA-FLSs, while STAT3 enhancement abrogated the effects (Fig. 6F, G). Western blot assay showed that overexpression of miR-671-5p reduced CyclinD1 and MMP9 protein levels in RA-FLSs, whereas STAT3 elevation reversed the effects (Fig. 6H). ELISA results showed that miR-671-5p overexpression decreased the concentrations of TNF-α and IL-1β in RA-FLSs, but the effects were weakened by upregulating STAT3 (Fig. 6I). Thus, we concluded that miR-671-5p overexpression suppressed RA-FLS development by targeting STAT3.

### Circ_0001947 knockdown suppressed STAT3 expression by targeting miR-671-5p

**Fig. 7:**
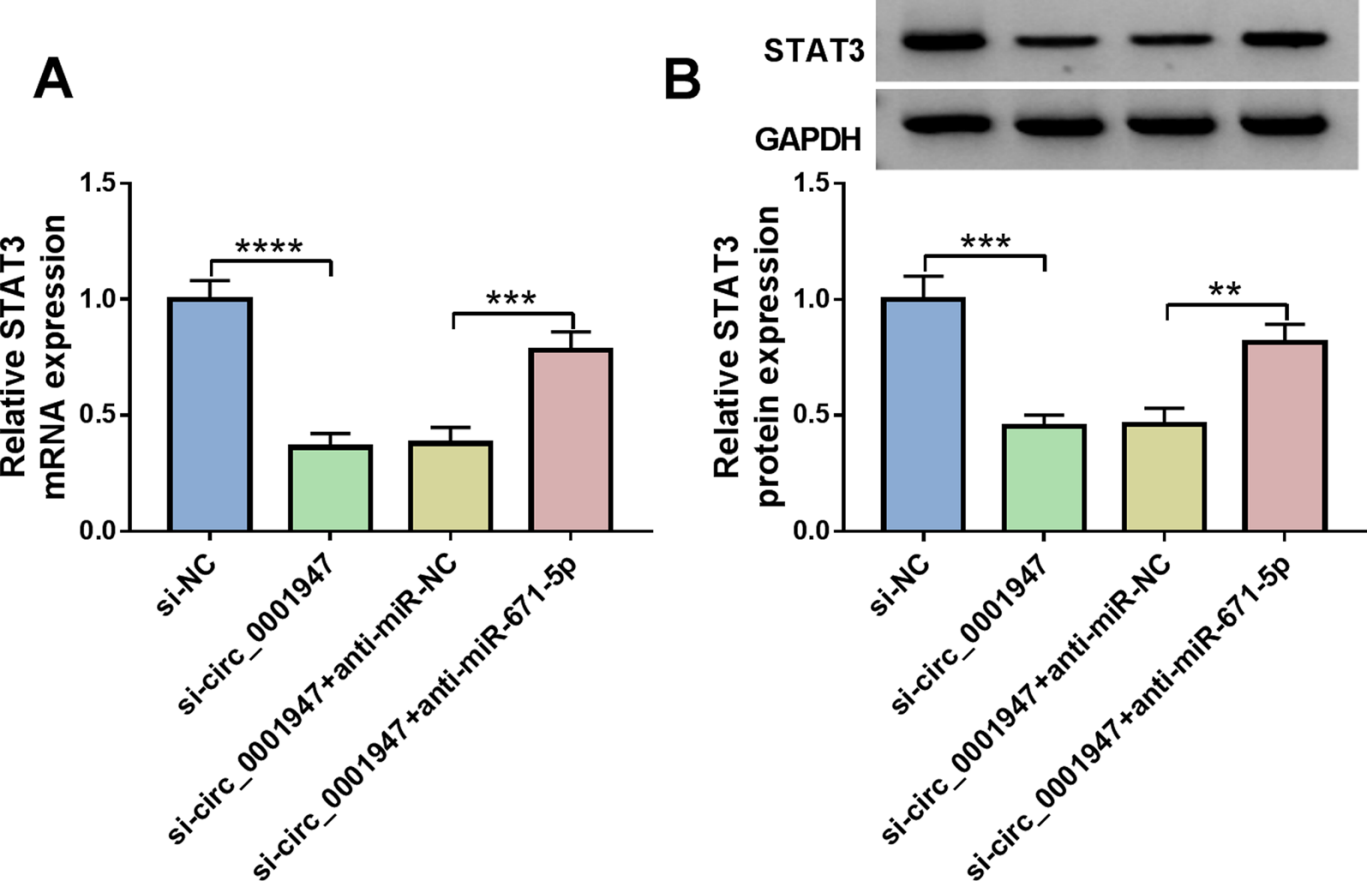
Circ_0001947 directly targeted miR-671-5p to regulate STAT3 expression in RA-FLSs. **A**, **B** After RA-FLSs were transfected with si-NC, si-circ_0001947, si-circ_0001947 + anti-miR-NC or si-circ_0001947 + anti-miR-671-5p, the mRNA and protein levels of STAT3 in RA-FLSs were examined by qRT-PCR and western blot, respectively. **P < 0.01, ***P < 0.001, ****P < 0.0001

At last, the relationships among circ_0001947, miR-671-5p and STAT3 were further analyzed. It was found that circ_0001947 silencing decreased STAT3 mRNA and protein levels in RA-FLSs, whereas miR-671-5p inhibition reversed the effects (Fig. 7A, B).

## Discussion

CircRNAs have been found to play an important role in the immune system and are closely associated with the progression of autoimmune diseases, including RA [36]. However, the roles of circRNAs in RA are not broadly elucidated. In this study, we identified that circ_0001947 contributed to RA-FLS processes via a novel regulatory pathway of miR-671-5p/STAT3.

Previous studies have shown that the dysregulation of circ_0001947 is linked to the development of NSCLC [2], gastric cancer [4] and acute myeloid leukemia [12]. Moreover, Qu et al. and Zhi et al. demonstrated that circ-AFF2 is highly expressed in RA and aggravated RA-FLS growth, inflammation, invasion and migration via regulating miR-650/CNP axis and miR-375/TAB2 axis [23, 33]. Correspondingly, the overexpression of circ_0001947 was observed in the synovial tissues and FLSs of RA. Functionally, circ_0001947 deficiency repressed the growth, invasion, migration and inflammatory response and promoted the apoptosis in RA-FLSs. Our findings further demonstrated the promotional effect of circ_0001947 on RA.

Currently, the involvement of circRNA/miRNA/mRNA axis in RA has been gradually identified [13, 18, 34]. Thus, we further explored the potential mechanisms of circ_0001947 in RA-FLS processes. Circ_0001947 was discovered to serve as the sponge for miR-671-5p. Chen et al. unraveled that miR-671-5p inhibited cell viability, inflammation and invasion via circ-PTTG1IP/miR-671-5p/TLR2 pathway [5]. Ma et al. manifested that miR-671-5p was related to RA-FLS malignant changes by circ-FAM120A/miR-671-5p/MDM4 pathway [18]. Herein, we demonstrated that circ_0001947 silencing inhibited the dysfunction and inflammation of RA-FLSs by targeting miR-671-5p. Furthermore, overexpression of miR-671-5p inhibited RA-FLS growth, invasion, migration and inflammation and induced apoptosis by interacting with STAT3, which was reported to influence RA progression via acting as the target of miR-20a [31], miR-301a-3p [29] and miR-140-5p [37].

However, there were still some limitations in this study. For example, the tissue samples were limited. The in vivo experiments were lacking to verify our conclusions.

To our best knowledge, we performed experiments using RA-FLSs to verify the functions of circ_0001947 in RA. We found that circ_0001947 was overexpressed in RA-FLSs. Moreover, we firstly confirmed the participation of circ_0001947/miR-671-5p/STAT3 in RA-FLS processes, including proliferation, invasion, migration, apoptosis and inflammation. Our findings provided a novel insight into RA pathogenesis and the reduction of circ_0001947 level might be a novel avenue for RA treatment.

## Data Availability

Not applicable.

## Abbreviations

RA: Rheumatoid arthritis
FLSs: Fibroblast-like synovial cells
circRNAs: Circular RNAs
ncRNAs: Non-coding RNAs
miRNAs: MicroRNAs
STAT3: Signal transducer and activator of transcription 3
